# Association of Apathy with Poor Sleep Quality in Patients with Early Parkinson’s Disease

**DOI:** 10.3390/medicina61111906

**Published:** 2025-10-24

**Authors:** Hak-Loh Lee, Seong-Min Choi, Soo Hyun Cho, Byeong C. Kim

**Affiliations:** 1 Department of Neurology, Chonnam National University Hospital, Gwangju 61469, Republic of Korea; ss10004ok@naver.com (H.-L.L.); k906141h@hanmail.net (S.H.C.); byeong.kim7@gmail.com (B.C.K.); 2 Department of Neurology, Chonnam National University Medical School, Gwangju 61469, Republic of Korea

**Keywords:** Parkinson’s disease, apathy, sleep

## Abstract

*Background and Objectives*: Apathy and sleep problems are frequently observed among non-motor symptoms (NMSs) in Parkinson’s disease (PD), but the relationship between the two has not been well investigated. This study aimed to determine the extent to which apathy and sleep disturbances are present in people with early PD and whether apathy affects sleep quality. *Materials and Methods*: Patients diagnosed with early PD, defined as modified Hoehn and Yahr (mHY) stages 1-3 and a disease duration of no more than 5 years, were included in the study. Demographic characteristics were collected, and motor and NMSs including apathy and sleep disturbance were investigated with relevant scales. *Results*: Of 302 patients with PD, apathy was found in 97 (32.1%) patients. Patients with apathetic PD had significantly less formal education, a more advanced mHY stage, and higher scores on the Unified Parkinson’s Disease Rating Scale (UPDRS) part II, total Non-Motor Symptom Scale (NMSS), Beck Depression Inventory (BDI), Apathy Evaluation Scale (AES), and Pittsburgh Sleep Quality Index (PSQI) global scores than patients with non-apathetic PD. The PSQI global score showed significant associations with years of education, UPDRS-II, total NMSS, Mini-Mental State Examination, BDI, and AES scores. For each component of the PSQI, only sleep latency was different between patients with apathetic and non-apathetic PD. Partial correlation analyses for determining the association between apathy and sleep disturbance revealed a significant positive correlation. *Conclusions*: Apathy is common and associated with poor sleep quality in patients with early PD. These findings suggest that recognizing and addressing apathy may be relevant for managing sleep disturbances in this population.

## 1. Introduction

Apathy refers to diminished motivation that cannot be attributed to cognitive impairment, emotional distress, or altered consciousness [1], is a frequent neuropsychiatric symptom in Parkinson’s disease (PD) [2,3]. Its prevalence varies across diagnostic methods [4], but it is consistently associated with greater functional impairment, poorer quality of life, and caregivers’ emotional distress [5,6]. Sleep disturbances are also highly prevalent in PD, affecting up to 70% of patients [7]. These encompass insomnia, restless legs syndrome, daytime hypersomnolence, and rapid eye movement (REM) sleep behavior disorder (RBD) [8], and are linked to cognitive decline and reduced quality of life [9,10].

Evidence suggests a possible link between sleep and apathy: circadian rhythm disruption has been associated with apathy after stroke [11], and PD patients with RBD report more frequent apathy symptoms than those without [12]. However, little is known about whether apathy influences sleep quality in PD. While apathy and depression often coexist in PD, they are regarded as distinct clinical constructs. Apathy is primarily characterized by diminished motivation and goal-directed behavior, whereas depression is defined by pervasive negative mood and affective symptoms [13,14,15,16]. The substantial overlap between the two conditions can complicate clinical assessment, underscoring the need for refined measures to clearly distinguish these syndromes [13,14,15,16,17].

This research sought to determine the prevalence of apathy and sleep-related problems among patients with early PD and to examine whether apathy is associated with poor sleep quality. To assess these symptoms, we employed the Apathy Evaluation Scale (AES), which is widely validated for detecting apathy in PD, and the Pittsburg Sleep Quality Index (PSQI), a standard tool for evaluating subjective sleep quality. In addition, depressive symptoms were evaluated using the Beck Depression Inventory (BDI), though distinguishing them from apathy remains a clinical challenge.

## 2. Materials and Methods

### 2.1. Study Population and Collection of Clinical Data

Patients with early PD who attended our outpatient clinic were recruited for the study. The diagnosis of PD was made according to the Movement Disorder Society (MDS) clinical diagnostic criteria for PD [18], which was confirmed by a movement disorder specialist (SM Choi). Included patients had modified Hoehn and Yahr (mHY) stages [19] ranging from 1 to 3 and a disease duration ≤5 years. Exclusion criteria included dementia, the presence of non-PD neurodegenerative disease, and clinically relevant abnormalities on brain magnetic resonance imaging. Approval for this study was obtained from the institutional review board of our hospital, and all procedures followed the ethical standards of the 1964 Helsinki Declaration.

Neurologists (HL Lee and SH Cho), who had been trained in advance for the survey, conducted patient interviews and examinations. Patients’ demographic characteristics such as age, sex, disease duration, formal education level, and BMI (Body Mass Index) were collected. Disease severity and motor function were assessed using the mHY stages (ranging from 1 to 5; higher stages indicate more severe motor impairment) and parts II and III of the Unified Parkinson’s Disease Rating Scale (UPDRS-II and UPDRS-III) [20]. UPDRS-II (0–52 points) evaluates activities of daily living (ADLs), and UPDRS-III (0–108 points) assesses motor symptoms, with higher scores indicating greater impairment. Non-motor symptoms (NMSs) were evaluated using the Korean version of the Non-Motor Symptom Scale (K-NMSS) [21], which comprises 30 items across 9 domains (cardiovascular, sleep/fatigue, mood/cognition, perceptual problems, attention/memory, gastrointestinal, urinary, sexual function, and miscellaneous). Scores range from 0 to 360, with higher scores indicating more severe non-motor symptoms. Cognitive performance was measured using the Korean Mini-Mental State Examination (K-MMSE, 0–30 points), with lower scores indicating poorer cognitive function [22]. Depression was measured using the Beck Depression Inventory (BDI, 0–63 points) [23], with higher scores indicating more severe depressive symptoms. The assessment of apathy was conducted using the Korean version of the Apathy Evaluation Scale (K-AES, 18–72 points) [24,25]. A threshold score of 38/39 on the scale was applied to differentiate patients with apathy from those without, with higher scores indicating greater apathy. The evaluation of sleep disturbances was conducted using the Korean version of the Pittsburgh Sleep Quality Index (PSQI, 0–21 points) [26,27]. Higher scores indicate poorer sleep quality.

### 2.2. Data Analysis

Independent sample t-tests were used to compare continuous variables, and chi-square tests were applied for categorical variables when analyzing clinical differences between apathetic and non-apathetic PD patients. Pearson correlation analysis was performed to explore potential factors affecting the PSQI score. Scores of each PSQI component between apathetic and non-apathetic PD were compared by ANCOVA (adjusted for formal education, mHY stage, and UPDRS-II scores). Partial correlation analysis was conducted to assess the relationship between apathy and sleep disturbance while excluding significant factors from the previous tests. Covariates such as education level, mHY stage, and UPDRS-II scores were included because these variables significantly differed between apathetic and non-apathetic groups and are known to influence both NMSs and sleep quality in PD. The sample size was determined based on the number of eligible patients during the study period and provides sufficient power for the conducted analyses, although future prospective studies with formal power calculation are warranted. All statistical analyses were carried out using SPSS version 27.0 (IBM Corp.; Armonk, NY, USA), and results with P value < 0.05 were deemed significant.

## 3. Results

A total of 302 PD patients were included, comprising 121 men and 181 women, with an average age of 67.5 ± 9.4 years, mean disease duration 18.0 ± 16.9 months, and an average formal education of 8.8 ± 4.7 years. Apathy was observed in 97 of the PD patients, representing 32.1% of the cohort. Age, sex, disease duration, and BMI did not differ significantly between PD patients with and without apathy. Patients with apathetic PD had significantly less formal education and a higher mHY stage and UPDRS-II score than those with non-apathetic PD. The UPDRS-III scores did not differ significantly between the groups. In terms of non-motor variables, patients with apathetic PD had significantly higher NMSS total, BDI, AES, and PSQI global scores than patients with non-apathetic PD, even after statistically adjusting for formal education level, mHY stage, and UPDRS-II score. The MMSE scores did not differ significantly between the groups (Table 1).

Pearson correlation analysis revealed significant associations between the PSQI global score and formal education level, as well as UPDRS-II, NMSS total, MMSE, BDI, and AES scores. No correlations were observed between the PSQI global score and age, disease duration, BMI, or UPDRS-II score (Table 2).

For each component of the PSQI, we analyzed whether there was a difference between patients with apathetic and non-apathetic PD. After controlling for formal education level, mHY stage, and UPDRS-II score, the sole significant difference between the two groups was in sleep latency (Table 3).

To determine the association between apathy and sleep disturbances, partial correlation analyses were conducted. After adjusting for demographic factors (age, sex, disease duration, formal education level, and BMI), the PSQI global score showed a significant positive correlation with the AES score. This relationship remained statistically significant even after adding adjustments for the mHY stage, UPDRS part II and III scores, as well as MMSE scores. However, when the BDI score was further adjusted, the association between the two was not statistically significant (Table 4).

## 4. Discussion

This study examined the association between apathy and sleep disturbances in early PD patients and compared various sleep components between those with and without apathy. Apathy was common in our cohort of patients with PD, and patients with apathetic PD exhibited significantly higher mHY stages and elevated UPDRS-II, total NMSS, BDI, and PSQI global scores compared to those without apathy. The PSQI global score showed significant associations with formal education level, as well as UPDRS-II, total NMSS, MMSE, BDI, and AES scores. Of each of the PSQI components, only sleep latency showed significant differences between patients with apathetic and non-apathetic PD. Furthermore, apathy in PD patients was found to be significantly linked to sleep disturbances, even after adjusting for various clinical factors excluding depression.

Apathy is one of the most common NMSs, with a prevalence of 20–36% in de novo PD, depending on the diagnostic method [4,28]. The prevalence of apathy tends to decrease with dopaminergic treatment in patents with early PD [4]. However, when the duration of PD is 5–10 years, the prevalence of apathy increases to 40% in those without dementia and 60% in those with dementia [4]. Of the 302 patients in our study population, 97 (32.1%) had apathy, which is similar to prior studies of patients with untreated PD.

In previous reports, patients with apathetic PD tended to be older and have less formal education, an extended disease duration, greater motor symptom severity, worse cognitive function, and higher depression scores than patients with non-apathetic PD [4,17,29]. Our study also showed a higher mHY stage and UPDRS-II score in patients with apathetic PD. This finding may lend support to the hypothesis that dysfunction of the nigrostriatal pathway contributes to the development of apathy [21]. However, the UPDRS-III score was not significantly different between the two groups, suggesting that apathy is not a fully dopamine-dependent symptom [13,17]. MMSE scores did not differ significantly between the two groups; this may be because our study included patients with early PD and excluded those with dementia.

In our study, the PSQI global score was positively correlated with the UPDRS-II and NMSS total scores and negatively correlated with the MMSE score. These results are consistent with the previous reports of higher ADL and NMSs scores in patients with PD with sleep disturbances [30,31]. The PSQI global score was also positively correlated with the BDI and AES scores. These observations align with prior research demonstrating an association between disturbed nocturnal sleep and both depression and apathy [32]. As the substantia nigra in the mesencephalon is connected with the subthalamic nuclei and basal ganglia, alterations in the direct and indirect pathways in PD patients may underlie this association, linking apathy with disrupted sleep regulation [33,34].

Sleep disturbances are common in patients with PD. Sleep architecture in early PD is typically marked by increased latency, reduced efficiency, a higher proportion of stage 1 sleep, and decreased REM sleep [35]. In our study, apathy in PD was found to have a significant effect on sleep latency among the PSQI components. One previous study has shown that the PD duration is associated with sleep latency [36], but there are no reports on how apathy affects sleep latency. An actigraphy-based study investigating the effect of sleep quality on cognition in PD patients reported that sleep latency was significantly associated with disease duration, attention, and executive function [9]. In another actigraphy-based study of sleep patterns in patients with AD, another common neurodegenerative disorder, the relationship between apathy and sleep latency was not statistically significant [37].

The partial correlation analysis showed a positive correlation between the PSQI global score and AES score; this correlation remained statistically significant even after adjustments for demographic factors (age, sex, disease duration, formal education level, and BMI) and PD-related clinical variables (mHY, UPDRS-III, UPDRS-II, MMSE). However, this was not the case when the scale for depression was included as a covariate. Although many studies have identified apathy and depression as distinct symptoms [13,14,15,16,17], the measurement tools used in this study may not completely disentangle them. Both the AES and BDI contain overlapping items, which may lead to inflated scores in patients presenting with mixed motivational and affective symptoms. Consequently, patients with apathetic PD may also present with higher BDI scores than those without apathy, partially explaining why the association between apathy and sleep disturbance was diminished after adjusting for depression. This limitation underscores the difficulty of separating motivational deficits from affective symptoms in PD. Further research should aim to clarify the relationship between pure apathy and sleep disturbance, ideally through multidimensional assessment tools, DSM-5-based clinical interviews, and more objective sleep measures. These findings also suggest that early recognition of apathy in PD, particularly when accompanied by sleep disturbances, could be clinically useful for monitoring patients and informing therapeutic strategies aimed at improving overall quality of life. From a practical perspective, incorporating routine apathy screening into the management of PD patients presenting with sleep disturbances may help to identify those at risk more quickly and encourage more integrated, multidisciplinary care strategies.

Beyond clinical correlations, there are several neurobiological mechanisms that may underlie the relationship between apathy and sleep disturbance in PD. Apathy has been strongly linked to dysfunction of mesocorticolimbic and frontostriatal circuits, particularly the anterior cingulate cortex, ventromedial prefrontal cortex, and nucleus accumbens, which are modulated by dopaminergic signaling [38]. These dopaminergic circuits are not only central to motivational drive, but also interact with hypothalamic and brainstem nuclei that regulate circadian rhythmicity and sleep–wake transitions [39]. Furthermore, serotonergic and noradrenergic projections to the hypothalamus and suprachiasmatic nucleus (SCN) have been shown to contribute to both motivational processing and circadian regulation, suggesting the presence of shared neurochemical substrates [40]. Circadian misalignment, facilitated by degeneration of the SCN and its dopaminergic and retinal inputs, has been linked to adverse outcomes including impaired reward processing, mood changes, and reduced goal-directed behavior [39,41]. Collectively, these overlapping pathways provide a plausible biological explanation for the observed association between apathy and poor sleep quality in early PD, emphasizing the importance of integrated therapeutic approaches targeting both motivational and circadian systems.

## 5. Limitations and Future Directions

This study has several limitations. A primary limitation of this study is its retrospective design, which may introduce selection and information bias because it relies on pre-existing clinical records. To minimize these issues, we applied strict inclusion and exclusion criteria, employed a standardized data extraction form, and blinded the data extractors to participants’ clinical outcomes. In addition, we attempted to reduce potential confounding by adjusting for major covariates in the statistical analysis. Despite these efforts, residual bias cannot be fully excluded, and the results should therefore be interpreted with caution. Sleep quality was assessed only through subjective measures (PSQI) without objective validation, which may have introduced bias. The absence of a control group precluded comparison of apathy prevalence and its relationship with sleep disturbance to a control population. Although apathy and depression are distinct entities, the scales used (AES, BDI) share overlapping constructs, potentially limiting phenotypic precision and confounding the results. The cross-sectional design prevents causal inference, and we lacked detailed medication histories, including antidepressant use, and did not assess possible sleep deprivation prior to the hospital evaluation, both of which may have influenced the findings. Future studies should include objective assessments such as polysomnography and actigraphy, employ longitudinal designs, incorporate multidimensional apathy assessments, and apply refined DSM-5-based approaches to better elucidate the independent role of apathy in sleep disturbances and its distinction from mood symptoms.

## 6. Conclusions

In this study, apathy was common and was associated with poor sleep quality in patients with early PD. These findings suggest that identifying and managing apathy may be relevant for addressing sleep disturbances in this population. However, given the potential confounding effects of depressive symptoms, these findings should be interpreted with caution. To clarify whether apathy directly contributes to poor sleep quality and to better understand its clinical implications, longitudinal, multimodal studies incorporating refined apathy measures and objective sleep metrics are warranted.

## Figures and Tables

**Table 1:** Clinical features of Parkinson’s disease patients with and without apathy.

	No Apathy (n = 205)	Apathy (n = 97)	*p*-Value	ANCOVA *p*-Value
Age (years)	67.1 ± 9.5	68.8 ± 9.2	0.155	
Sex (male:female)	85:120	36:61	0.530	
Disease duration (months)	16.8 ± 16.5	20.3 ± 18.1	0.097	
Formal education (years)	9.1 ± 4.5	7.9 ± 4.9	0.048	
BMI (kg/m^2^)	23.7 ± 3.1	23.2 ± 3.1	0.232	
mHY stage	1.8 ± 0.6	2.1 ± 0.8	0.002	
UPDRS-III score	21.0 ± 10.0	23.0 ± 10.3	0.111	
UPDRS-II score	5.3 ± 4.6	10.5 ± 8.0	<0.001	
NMSS total score	37.5 ± 26.3	70.6 ± 38.5	<0.001	<0.001
MMSE score	26.3 ± 3.2	25.6 ± 3.6	0.094	0.844
BDI score	8.1 ± 7.0	18.8 ± 11.6	<0.001	<0.001
AES score	30.1 ± 5.9	47.1 ± 6.2	<0.001	<0.001
PSQI global score	5.9 ± 3.5	8.3 ± 4.0	<0.001	0.011

Data are presented as the mean ± standard deviation. The covariates of ANCOVA were formal education level, mHY stage, and UPDRS-II scores. ANCOVA, analysis of covariance; BMI, Body Mass Index; mHY, modified Hoehn and Yahr; UPDRS, Unified Parkinson’s Disease Rating Scale; NMSS, Non-Motor Symptoms Scale; MMSE, Mini-Mental State Examination; BDI, Beck Depression Inventory; AES, Apathy Evaluation Scale; PSQI, Pittsburgh Sleep Quality Index.

**Table 2:** Factors associated with the Pittsburgh Sleep Quality Index global score.

	Correlation Coefficient	*p*-Value
Age (years)	0.074	0.200
Disease duration (months)	0.049	0.397
Formal education (years)	−0.162	0.007
BMI (kg/m^2^)	0.051	0.384
mHY stage	0.098	0.090
UPDRS-III score	0.049	0.403
UPDRS-II score	0.392	<0.001
NMSS total score	0.489	<0.001
MMSE score	−0.175	0.003
BDI score	0.498	<0.001
AES score	0.322	<0.001

BMI, Body Mass Index; mHY, modified Hoehn and Yahr; UPDRS, Unified Parkinson’s Disease Rating Scale; NMSS, Non-Motor Symptoms Scale; MMSE, Mini-Mental State Examination; BDI, Beck Depression Inventory; AES, Apathy Evaluation Scale.

**Table 3:** Pittsburgh Sleep Quality Index component scores in Parkinson’s disease patients with and without apathy.

	No Apathy (n = 205)	Apathy (n = 97)	*p*-Value	ANCOVA *p*-Value
Subjective sleep quality	1.13 ± 0.76	1.53 ± 0.86	<0.001	0.051
Sleep latency	1.21 ± 0.95	1.64 ± 0.98	<0.001	0.019
Sleep duration	1.01 ± 0.94	1.34 ± 1.11	0.014	0.170
Habitual sleep efficiency	0.54 ± 0.93	0.91 ± 1.20	0.009	0.191
Sleep disturbances	1.20 ± 0.45	1.43 ± 0.55	<0.001	0.065
Use of sleep medication	0.20 ± 0.70	0.34 ± 0.92	0.187	0.609
Daytime dysfunction	0.69 ± 0.91	1.11 ± 1.11	0.001	0.223

Data are presented as the mean ± standard deviation. The covariates of ANCOVA were formal education level, mHY stage, and UPDRS-II scores.

**Table 4:** Partial correlation analyses between the Pittsburgh Sleep Quality Index global score and the Apathy Evaluation Scale score.

	*r*	*p*-Value
Age, sex, duration, education, BMI	0.271	<0.001
adding mHY, UPDRS-III, UPDRS-II	0.146	0.019
adding MMSE	0.151	0.019
adding BDI	0.002	0.975

BMI, Body Mass Index; mHY, modified Hoehn and Yahr; UPDRS, Unified Parkinson’s Disease Rating Scale; MMSE, Mini-Mental State Examination; BDI, Beck Depression Inventory.

## Data Availability

Anonymized data is available upon request from the corresponding author.
